# Temporal Validation of a Predictive Score for Death in Children with Visceral Leishmaniasis

**DOI:** 10.1155/2021/6688444

**Published:** 2021-12-22

**Authors:** Juliana Foinquinos, Maria do Carmo Duarte, Jose Natal Figueiroa, Jailson B. Correia, Nara Vasconcelos Cavalcanti

**Affiliations:** ^1^Instituto de Medicina Integral Prof. Fernando Figueira, Recife, Brazil; ^2^Universidade de Pernambuco, Recife, Brazil; ^3^Faculdade Pernambucana de Saúde, Recife, Brazil

## Abstract

**Objectives:**

To perform a temporal validation of a predictive model for death in children with visceral leishmaniasis (VL).

**Methods:**

A temporal validation of a children-exclusive predictive model of death due to VL (Sampaio et al. 2010 model), using a retrospective cohort, hereby called validation cohort. The validation cohort convenience sample was made of 156 patients less than 15 years old hospitalized between 2008 and 2018 with VL. Patients included in the Sampaio et al. 2010 study are here denominated derivation cohort, which was composed of 546 patients hospitalized in the same hospital setting in the period from 1996 to 2006. The calibration and discriminative capacity of the model to predict death by VL in the validation cohort were then assessed through the procedure of logistic recalibration that readjusted its coefficients. The calibration of the updated model was tested using Hosmer–Lemeshow test and Spiegelhalter test. A ROC curve was built and the value of the area under this curve represented the model's discrimination.

**Results:**

The validation cohort found a lethality of 6.4%. The Sampaio et al. 2010 model demonstrated inadequate calibration in the validation cohort (Spiegelhalter test: *p*=0.007). It also presented unsatisfactory discriminative capacity, evaluated by the area under the ROC curve = 0.618. After the coefficient readjustment, the model showed adequate calibration (Spiegelhalter test, *p*=0.988) and better discrimination, becoming satisfactory (AUROC = 0.762). The score developed by Sampaio et al. 2010 attributed 1 point to the variables dyspnea, associated infections, and neutrophil count <500/mm^3^; 2 points to jaundice and mucosal bleeding; and 3 points to platelet count <50,000/mm^3^. In the recalibrated model, each one of the variables had a scoring of 1 point for each.

**Conclusion:**

The temporally validated model, after coefficient readjustment, presented adequate calibration and discrimination to predict death in children hospitalized with VL.

## 1. Introduction

Visceral leishmaniasis (VL) is a systemic protozoan disease transmitted to humans by the bite of female phlebotomine sandflies infected with certain species of *Leishmania* [1]. It is endemic in 98 countries and territories worldwide, though 90 per cent of cases are reported from only 10 countries (Brazil, Ethiopia, Eritrea, India, Iraq, Kenya, Nepal, Somalia, South Sudan, and Sudan) [2, 3]. The World Health Organization (WHO) classifies VL as a neglected disease, as it occurs in poor regions, contributes to global inequality, and impairs the development of affected countries [2].

In Brazil, VL is present in 23 (out of 27) states, though 44.5% of cases reported come from the nine states that make the northeast region [4, 5]. Forty (40.9) per cent of cases occur in children aged 0–9 years [4, 5] and case fatality rate was up until recently on the rise nationwide, having reached 9.1% in 2019 [6].

The clinical presentation of VL is quite diverse. Most people develop either asymptomatic or oligosymptomatic forms. But some will present as a severe, systemic illness that will likely lead to death if untreated. Following an incubation period of about three to eight months, prolonged fever and splenomegaly will raise diagnostic suspicion in areas where VL is endemic. Symptoms will usually develop insidiously and can include hepatomegaly, cough, malaise, weight loss, and appetite loss [7, 8].

Factors associated with a poor prognosis and death by VL have been described in the medical literature, but few reports included children as patients and we found only two publications involving exclusively the pediatric population [9, 10]. A study conducted in Minas Gerais, Brazil, has shown that low platelet counts (<85,000/mm^3^), age younger than 18 months, and respiratory findings on examination were associated with poor prognosis and death [9]. On the other hand, in Tunisia, the risk of death was associated with bleeding on admission, white cell count <4,000/mm^3^, elevated aminotransferases, and delay between onset of symptoms and admission >15 days [10]. None of these studies, however, proposed a scoring system to identify children at greater risk for death by VL.

Prognostic scores based on risk factors for adverse outcomes and/or death by VL involving children are scarcely published [11–17] and we found only one study assessing exclusively the pediatric population [11]. A study conducted in Pernambuco, Brazil, by our research group (Sampaio et al., 2010) included 546 children and adolescents less than 15 years of age hospitalized with confirmed VL in the period from 1996 to 2006. We have found a 10% case fatality rate and the presence of dyspnea, associated infections, jaundice, bleeding gums, severe neutropenia (neutrophil count <500/mm^3^), and severe thrombocytopenia (platelet count <50,000/mm^3^) which were shown to be independent risk factors for death by VL. The presence of two or more factors increased the risk of death and a score ≥3 was selected as the best predictor of death as it was able to gather the most adequate combination of sensitivity (88.7%), specificity (78.5%), positive predictive value (32.0%), negative predictive value (78.5%), and area under ROC curve (AUROC) (89.5%) [11]. However, this study lacked an external validation of the model and did not allow to accommodate temporal changes in diagnosis and treatment of VL in recent decades which improved considerably and this may have had an effect on the severity of patients at hospital admission to centers of tertiary care. The present study aims to perform a temporal validation of the Sampaio et al. 2010 predictive model, assessing its applicability and accuracy over time.

## 2. Methods

A temporal validation of the Sampaio et al. 2010 VL predictive model was performed, using a retrospective cohort, denominated validation cohort, constituted by 156 patients less than 15 years old hospitalized between 2008 and 2018 with VL at the Professor Fernando Figueira Institute of Integrative Medicine (IMIP), Pernambuco, Northeast of Brazil. Patients included in the Sampaio et al. 2010 study are here denominated derivation cohort which was composed of 546 patients hospitalized in the period from 1996 to 2006 [11].

Once VL notification is compulsory in Brazil, patients considered eligible to participate in the validation cohort were identified from the notification registry of the epidemiology center of the hospital. Children in the registry had their medical records revised from admission to their outcome (hospital discharge, transfer, or death). Patients were included if VL was laboratory confirmed (positive Giemsa stain of bone marrow or spleen smear, enzyme-linked immunosorbent assay (ELISA), and immunofluorescence or rk39 rapid test) or if they had clinical-epidemiological criteria (patient coming from area with VL transmission with a favorable response to treatment). Patients with an alternative diagnosis (e.g. malignancies and prolonged septicemic enterobacterial disease) found during hospital investigations were excluded. Recurrences of VL were counted only once, and it was considered the last outcome for each patient.

For the validation cohort, a sample size calculation was not performed. Instead, a convenience sample of all patients less than 15 years old hospitalized with VL from the decade of 2008 to 2018 was selected. A total of 169 patients were identified, but 13 records were missing. Among the 156 included patients, there were 7 missing data, which represented a low percentage (4.5%) and therefore were excluded. There was no need to carry out any method of imputation, and thus a complete-case analysis was performed.

VL death was defined as hospital death as a consequence of VL and its complications. The occurrence of independent variables was considered when they were present at hospital admission and were defined as follows. Associated infection was considered when the physician judged the presence of any clinical sign suggestive of a community infection. Jaundice, edema, dyspnea, and petechiae were characterized on physical examination. Diarrhea was considered if either reported by the parents or observed by the physician. Mucosal bleeding was considered anywhere in the body such as epistaxis, bleeding gums, or upper digestive hemorrhage and could be either reported by the parents or observed by the physician.

The TRIPOD (transparent reporting of a multivariable prediction model for individual prognosis or diagnosis) Statement was used to guide the analysis and reporting of the study [18]. A 22-item checklist with the respective page for each item can be found as Supplementary Materials.

Once it was a retrospective study with analysis of medical records from the last two decades, a dismissal of the written informed consent was granted.

### 2.1. Statistical Analysis

Variables frequencies were compared in the two cohorts, using Pearson's chi-square test, as all quantitative variables had been categorized for statistical analysis.

The model developed by Sampaio et al., 2010, identified, through a multivariate logistic regression analysis, the following independent risk factors for death: dyspnea, associated infections, neutrophil count <500/mm³, jaundice, mucosal bleeding, and platelet count <50,000/mm³. The calibration (correct outcome probability for all levels of predicted risk) and discriminative capacity (score's capability to define different patient prognosis) of the model to predict death by VL in the validation cohort were then assessed through the procedure of logistic recalibration that readjusted its coefficients [19].

The calibration of the updated model was tested using Hosmer–Lemeshow test and Spiegelhalter test [20, 21]. A ROC curve was built and the value of the area under this curve represented the model's discrimination. Hosmer and Lemeshow [21] propose the following classification for the discriminative capacity of a predictive model related to the values of the AUROC: AUROC = 0.5 (absence of discrimination); 0.5 < AUROC < 0.7 (unsatisfactory discrimination); 0.7 ≤ AUROC < 0.8 (acceptable discrimination); 0.8 ≤ AUROC < 0.9 (excellent discrimination); and AUROC ≥ 0.9 (outstanding discrimination).

## 3. Results

The baseline characteristics of the validation cohort (*n* = 156) are presented in Table 1. Thirteen patients (7.2%) were considered as losses, as their medical records were not located. Among the assessed characteristics, a frequency around 65% of children less than 5 years of age was observed in the cohort. The lethality of the validation cohort was 6.4%. There was no statistically significant difference between the percentages of death in the cohorts (validation cohort 6.4% *vs.* derivation cohort 10.2%; *p*=0.198).

Initially, the discriminant capacity of the Sampaio et al. 2010 model applied to the validation cohort, measured by the AUROC, was 61.8% and considered unsatisfactory. In addition, the model's calibration was unsatisfactory, both for Hosmer–Lemeshow test (*p*=0.034: number of groups = 6) and Spiegelhalter test (*p*=0.007). Therefore, a logistical recalibration procedure was subsequently performed.

**Figure 1:**
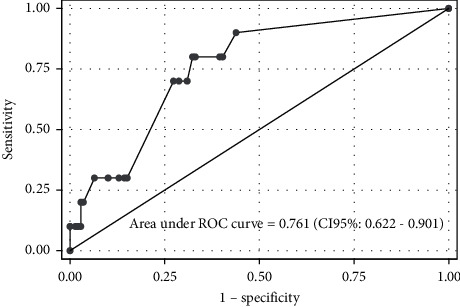
ROC curve obtained with the application of the recalibrated model to the validation cohort.

Table 2 presents the coefficients of the Sampaio et al. 2010 model that were obtained in the derivation cohort and recalibrated after the logistical recalibration procedure. After readjusting coefficients, the model presented adequate calibration (Spiegelhalter test: *p*=0.988) and its discrimination was considered satisfactory (AUROC = 0.762), as can be seen in Figure 1 that shows the ROC curve obtained with the application of the recalibrated model to the validation cohort. There was no statistically significant difference between the AUROC of the Sampaio et al. 2010 model and the recalibrated model (0.898 *vs.* 0.762, respectively; *p*=0.071).

The original prognostic score of Sampaio et al., 2010, and the proposed recalibrated score are described in Table 3. The score developed by Sampaio et al., 2010, attributed 1 point to dyspnea, associated infections, and neutrophil count <500/mm³; 2 points to jaundice and mucosal bleeding; and 3 points to platelet count <50,000/mm³. In the recalibrated model, each one of the variables had a scoring of 1 point, therefore any patient could have a score ranging from 0 to 6. A score ≥2 was selected as the most adequate predictor of death because it was able to congregate the most satisfactory combination of sensitivity (30%), specificity (81.3%), positive predictive value (10.2%), negative predictive value (94.2%), and area under ROC curve (55.6%).

## 4. Discussion

The present study proposed to temporally validate a predictive model for death in children with VL. It was hypothesized that changes and interventions that occurred over the years could have influenced the accuracy of the original cohort's score. The findings of the present study show that after coefficient readjustment, the Sampaio et al. 2010 model presented adequate calibration and discrimination to predict death in inpatient children with VL in Brazil.

The initial discriminative capacity of the validation cohort was considered unsatisfactory (61.8%), whereas in the Sampaio et al. 2010 study (derivation cohort), the discrimination was considered excellent (89.5%). This fact might have occurred due to various aspects. Models tend to discriminate better in the sample in which they are adjusted [20]. In the epidemiologic perspective, improvements in public health regarding healthcare for patients with VL in Pernambuco and Brazil were achieved during the past twenty years. One of these progresses was the decentralization of diagnosis and management of VL in Pernambuco state, by measures such as the availability of a rapid diagnostic test for VL (rK39) in the entire countryside of the state and the decentralization of treatment with liposomal amphotericin B [22, 23]. Additionally, the use of liposomal amphotericin B was amplified in Brazil in 2014 for children under one year of age and for severe cases of VL [24, 25]. Such measures allowed the early diagnosis and treatment of severe cases in lower complexity services, reducing the need to transfer patients to tertiary hospitals, which can be responsible for a reduced severity and lethality of patients. All these aspects could justify the need for recalibration of coefficients of the original cohort to optimize the score validation.

The current study evaluated clinical and laboratorial findings during the admission of patients to the hospital, and when the two cohorts were compared (data not shown), a significant decrease in the frequency of edema, dyspnea, bleeding gums, petechiae, and anemia in the admission and factors previously studied in the literature and known to be associated with death by VL were verified [9–17]. Such findings may be related to improvements in health conditions and decentralization of healthcare in the past two decades in Pernambuco state, as described above, what can justify the significant decrease in the frequency of the mentioned variables.

Bacterial infections and hepatic insufficiency represent the leading causes of death by VL in children [9, 11–17]. The main causes of death observed by Sampaio et al., 2010, and analyzed in the validation cohort (data not shown) are similar to those described in other studies, demonstrating that immediate causes of death persist over the years.

The score of the Sampaio et al. 2010 cohort was now temporarily validated and recalibrated in the present study, being able to be compared to other scores which would assess the pediatric population. We highlight that all other scores involving children also included adult patients [12–15] and one of them concluded that patients out of the pediatric age (more than 40 or 60 years) were factors associated with death by VL [14]. Another important association reported in some scores is the coinfection with HIV, a common finding in adults with VL, which is not commonly observed in the pediatric population [12, 14]. Previous studies described the coinfection VL-HIV in 4.5% and 14.3% of patients, all of them were adults [26, 27]. It was observed that patients coinfected with HIV were significantly older than patients without HIV (mean age of 36 for coinfected versus 14 for noninfected, *p* < 0.001) [27]. The present study and the one conducted by Sampaio et al., 2010, did not evaluate the occurrence of HIV coinfection.

Associated bacterial infection and jaundice were variables observed in all studies with pediatric patients found in the literature which developed a prognostic score [11–15]. Other variables found in the scores which exist in the literature and assessed in the present study include dyspnea [11, 12], lower age (less than 12 months [12] and less than 6 months [14]), edema [12, 14], elevation of aminotransferases [12], diarrhea [13], splenomegaly [14], and weight loss [14].

Despite the importance of the study, some limitations should be highlighted: once it is a retrospective cohort, it may exist lower precision in the registry of laboratorial and clinical data. The need for readjustment of coefficients of the Sampaio et al. 2010 model can also be considered a limitation, yet it can indicate adequacy to improvements in public health within the time interval between the two studies, once it was observed that patients were less severe at hospital admission and lethality decreased in relation to the derivation cohort.

The importance of the present study lies in the fact that a temporal validation was performed with a good discrimination after coefficient readjustment of the Sampaio et al. 2010 model, which is the only score that exclusively analyzed the pediatric population for death due to VL. The Sampaio's recalibrated score can be easily applied by health professionals of endemic areas, optimizing the identification of potentially severe cases and the subsequent adoption of earlier interventions such as the early and careful use of antimicrobials, transfusion of blood products, and transfer to intensive care units.

In conclusion, the temporally validated model, after coefficient readjustment, presented adequate calibration and discrimination to predict death in children hospitalized with VL. Improvements that occurred in the last 20 years in Brazil such as decentralization of management of these patients, early diagnosis through rK39 rapid test, and the expanded use of liposomal amphotericin B may have contributed to the decreased lethality in the pediatric population and to the need for coefficient readjustment. New prospective studies should be performed aiming to validate the present score in different pediatric populations.

## Figures and Tables

**Table 1:** Baseline characteristics of the validation cohort.

Characteristics^*∗*^	Validation cohort
	% (n/N)
Male sex	48.7 (76/156)
Age <5 years	65.4 (102/156)
Diarrhea	9.6 (15/156)
Edema	10.9 (17/156)
Jaundice	7.1 (11/156)
Dyspnea	9.0 (14/156)
Mucosal bleeding	0.6 (1/156)
Petechiae	1.9 (3/156)
Associated infections	12.8 (20/156)
Leukocyte <2,500/mm^3^	37.4 (58/155)
Platelets <50,000/mm^3^	28.8 (44/153)
Neutrophils <500/mm^3^	15.2 (23/151)
Hemoglobin <5 g/dL	11.0 (17/155)

^*∗*^Some characteristics did not have registry in the patient's medical records.

**Table 2:** Coefficients from the Sampaio et al. 2010 study obtained in the derivation cohort and recalibrated after procedure of logistical recalibration.

	Coefficient derivation cohort	Coefficient validation cohort recalibrated^*∗*^
Constant	−4.497735	−3.6151509
Dyspnea	1.015233	0.5304422
Associated infections	1.003439	0.5242800
Neutrophils <500/mm³	1.145403	0.5984538
Jaundice	1.490908	0.7789744
Mucosal bleeding	1.415629	0.7396424
Platelets <50,000/mm³	2.458888	1.2847277

^*∗*^Adjustment of the constant term: (−1.2651599) + 0.5224832 × (−4.497735) = −3.6151509. Adjustment of dyspnea coefficient: 0.5224832^*∗*^1.015233 = 0.5304422. Adjustment of associated infections coefficient: 0.5224832^*∗*^1.003439 = 0.5242800. Adjustment of neutrophil <500/mm³ coefficient: 0.5224832^*∗*^1.145403 = 0.5984538. Adjustment of jaundice coefficient: 0.5224832^*∗*^1.490908 = 0.7789744. Adjustment of mucosal bleeding coefficient: 0.5224832^*∗*^1.415629 = 0.7396424. Adjustment of platelets <50,000/mm³coefficient: 0.5224832^*∗*^2.458888 = 1.2847277.

**Table 3:** Sampaio's original prognostic score and recalibrated score after validation.

Variable	Original score	Recalibrated score
Dyspnea	1	1
Associated infections	1	1
Neutrophils <500/mm³	1	1
Jaundice	2	1
Mucosal bleeding	2	1
Platelets <50,000/mm³	3	1

## Data Availability

The original contributions presented in the study are included in the article and in the Supplementary Materials. Further inquiries can be directed to the corresponding author.

## Supplementary Materials

Supplementary Materials TRIPOD checklist: prediction model development and validation .
